# Efficacy and Safety of Empagliflozin on Nonalcoholic Fatty Liver Disease: A Systematic Review and Meta-Analysis

**DOI:** 10.3389/fendo.2022.836455

**Published:** 2022-02-24

**Authors:** Yuyuan Zhang, Xiaobo Liu, Huazhu Zhang, Xuechang Wang

**Affiliations:** ^1^College of Pharmacy, Dali University, Dali, China; ^2^Department of Pharmacy, Kunming Fourth People’s Hospital, Kunming, China

**Keywords:** NAFLD, empagliflozin, medication, meta-analysis, systematic review

## Abstract

**Objective:**

Clinical trials have recently shown a connection between nonalcoholic fatty liver disease (NAFLD) and empagliflozin. This paper aimed at comprehensively assessing the effectiveness and security of empagliflozin in NAFLD patients.

**Methods:**

PubMed, Embase, Web of Science, Cochrane Library, CNKI, CBM, Wan-Fang digital database, VIP, and WHO ICTRP were searched for randomized controlled trials (RCTs) on the role of empagliflozin in NAFLD from inception to November 2, 2021. For continuous dating, we used values of mean differences (MD) to present.

**Results:**

A total of four articles involving 244 NAFLD patients were included. Compared with the control group, empagliflozin could significantly reduce the body mass index (BMI) (MD: −0.98 [95% CI: −1.87, −0.10], *p* = 0.03), liver stiffness measurement (LSM) (MD: 0.49 [95% CI: −0.93, −0.06], *p* = 0.03), aspartate aminotransferase (AST) (MD: −3.10 [95% CI: −6.18, −0.02], *p* = 0.05), homeostasis model assessment of insulin resistance (HOMA-IR) (MD: −0.45 [95% CI: −0.90, 0.00], *p* = 0.05) of the treatment group.

**Conclusions:**

Empagliflozin can improve body composition, insulin resistance, and liver fibrosis and decrease the hepatic enzymes in patients with NAFLD. Empagliflozin emerges as a new option for treating patients with NAFLD. However, further research shall determine the efficacy and safety of empagliflozin in NAFLD.

## Introduction

Nonalcoholic fatty liver disease (NAFLD) is an often neglected etiology of chronic liver disease that is prevalent globally, resulting from the liver fatty acid accumulation and fibrosis without overmuch alcohol consumption (1). Nonalcoholic fatty liver (NAFL) and nonalcoholic steatohepatitis (NASH) are the two major subtypes of NAFLD. The patients diagnosed with NASH can subsequently progress to liver fibrosis, which increases the risks of cirrhosis and liver cancer (2, 3). NAFLD is also a dangerous factor that obviously grows the risk of cardiovascular and chronic kidney disease (4, 5). The prevalence of NAFLD is approximately 25% worldwide; furthermore, it is estimated that NAFLD shall be the most common index for liver transplantation by 2030 (6, 7).

The pathogenesis of NAFLD is complex, and several factors have been involved, including Western diet, lifestyle, and genetic susceptibility. NAFLD is most closely related to the metabolic environment, such as insulin resistance, abnormal lipid profile, and metabolic dysfunction, which is also why it has an appropriate new nomenclature as MAFLD (8, 9). Additionally, increased risk of more severe NAFLD is strongly associated with type 2 diabetes (T2DM) and diabetes-related complications (10). Currently, no available drug has been approved for the management of NAFLD (11). Although the insulin sensitizer pioglitazone has been proven to enhance metabolism and liver histology among NAFLD patients, it has limitations in clinical use due to significant adverse events including weight gain, lower extremity edema, and heart failure (12, 13).

Empagliflozin, a sodium-glucose cotransporter 2 (SGLT-2) inhibitor, is a novel oral hypoglycemic agent that inhibits glucose reabsorption, improving insulin resistance, leading to downregulation of SREBP-1c and blockage of new hepatic lipogenesis, thereby improving lipid metabolisms (14, 15). Currently, it has been shown that controlled clinical trials (RCTs) have evaluated the role of empagliflozin in NAFLD patients. However, the role of empagliflozin in NAFLD patients is controversial, and there is no comprehensive analysis on RCTs of empagliflozin. Hence, this research aiming to investigate the effectiveness and security of empagliflozin in NAFLD was made.

## Methods

### Data Sources and Search Strategy

This study was designed based on Preferred Reporting Items for conducting Systematic Reviews and Meta-Analyses (PRISMA) (16). The agreement of this research was registered with PROSPERO.

We conducted an electronic search in the databases below: Pubmed, Embase, Web of Science, Cochrane Library, China National Knowledge Internet (CNKI), Chinese Biomedical Literature Database (CBM), Wan-Fang digital database, China Science and Technology Journal Database (VIP), and WHO International Clinical Trial Registration Platform (ICTRP). The subject terms were as below: “NAFLD,” “empagliflozin”, and “randomized”. There are no language restrictions, and the last search was made on November 2, 2021. The detailed description of search measure is provided in **Supplementary Material 1**.

### Selection and Eligibility Criteria

The search results were screened by two reviewers for title, abstract, and full text, and the differences were resolved by a third reviewer. Inclusion criteria include RCTs on the effectiveness of empagliflozin in the treatment of NAFLD patients. Only original articles were included. Exclusion criteria were as follows: studies with nonhuman subjects, non-RCTs, systematic reviews, or meta-analyses.

### Data Extraction and Outcomes

Reviewers independently extracted relevant data from each study into a predesigned Excel spreadsheets, which included country of origin, year of publication, first author, trial design, inclusion criteria, study duration, study population, intervention and duration, participant gender and age, baseline patient information, and treatment outcomes. The outcomes included are biological indicators of liver function such as: aspartate transaminase (AST), alanine transaminase (ALT), low-density lipoprotein (LDL), high-density lipoprotein (HDL), and triglycerides (TG). Body composition includes the following: body weight and body mass index (BMI). Glycemic indices are as follows: fasting blood sugar (FBS), insulin, glycosylated hemoglobin A1c (HbA1C), and homeostatic model assessment of insulin resistance (HOMA-IR). Hepatic steatosis and fibrosis indicators are controlled attenuation parameter (CAP), liver stiffness measurement (LSM), NAFLD fibrosis score (NFS), and fibrosis-4 Index (FIB-4 index). For continuous dating, we extracted the means and standard deviation (SD). In the absence of the mean and SD, the data were transformed based on the existing formulae. Differences were resolved independently by a third reviewer.

### Statistical Analysis and Quality Assessment

For results, this meta-analysis selected mean difference (MD) to assess continuous variables and presented with a 95% CI. The degree of heterogeneity was quantified with the *I*^2^ statistic. *I*^2^ values of 25%, 50%, and 75% represented low, medium, and high heterogeneity, respectively. Random effects models were used to pool measures in all studies. *p*-values equal or less than 0.05 set as the cutoff for statistical significance. The quality of the RCT was evaluated using the Cochrane Risk of Bias Assessment Tool, including the following six criteria: random sequence generation, allocation concealment, blinding of patients, trialists, blinding of result assessors, incomplete result information, selective reporting, and other biases. Each item was assessed for risk of bias as “low risk”, “high risk”, or “unclear risk” according to the recommendations of the Cochrane Handbook. STATA 16 and RevMan 5.4 were used for statistical analysis and quality assessment.

## Results

### Search Results

**Figure 1:**
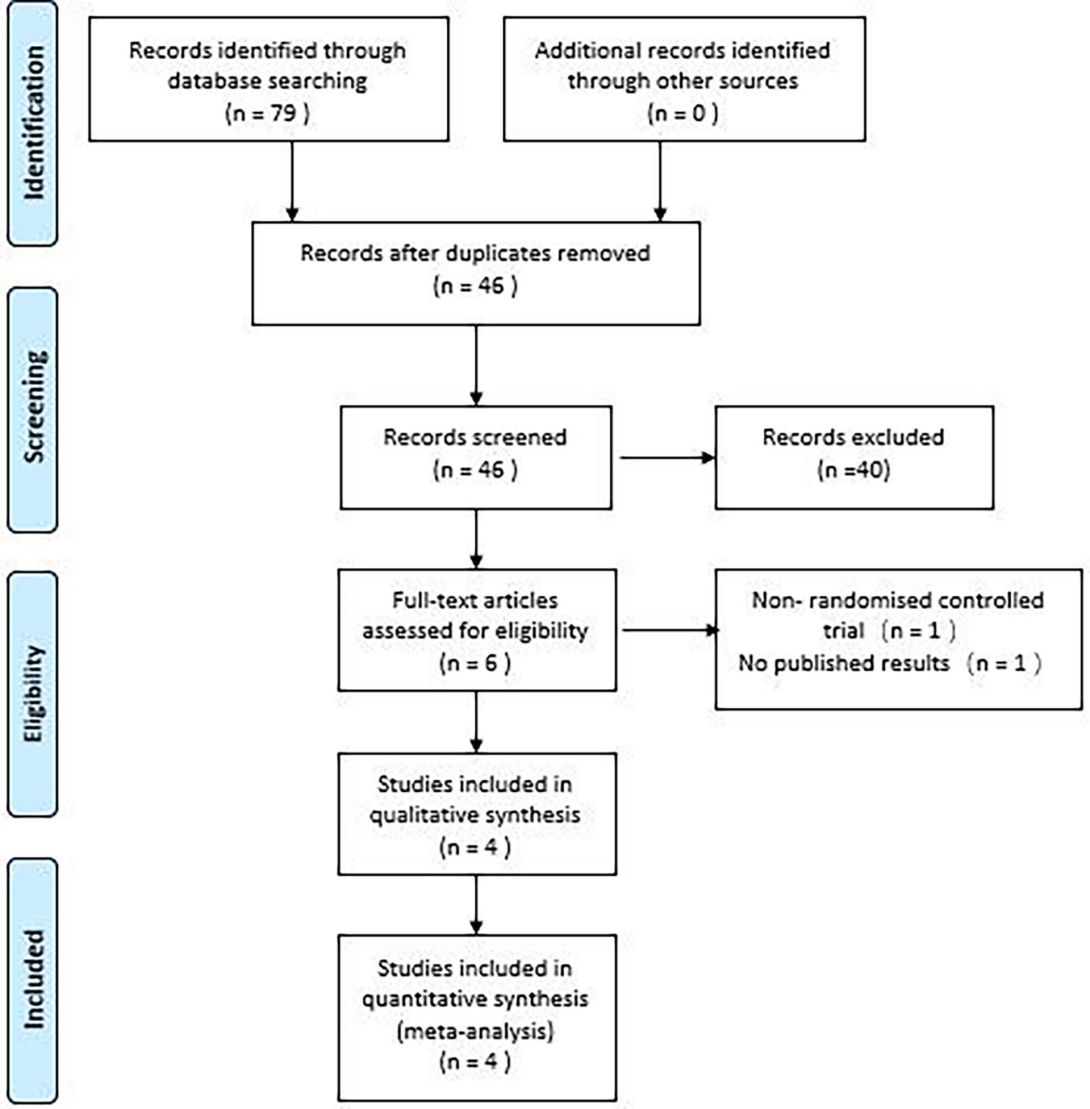
PRISMA flow chart.

The study initially identified 79 articles, which included 7 studies from PubMed, 18 from the Cochrane Library, 28 from Embase, 20 from the Web of Science, and 2 each from SINOMED, CNKI and Wanfang digital database, respectively. After excluding 33 duplicates, reviewing 46 titles and abstracts, 40 outcomes were excluded, and the remaining 6 outcomes were reviewed in full, resulting in 4 randomized controlled studies of empagliflozin for NAFLD included in the meta-analysis (17–20). The PRISMA flow chart (**Figure 1**) illustrates the detailed selection process.

### Study Characteristics and Quality Assessment

**Figure 2:**
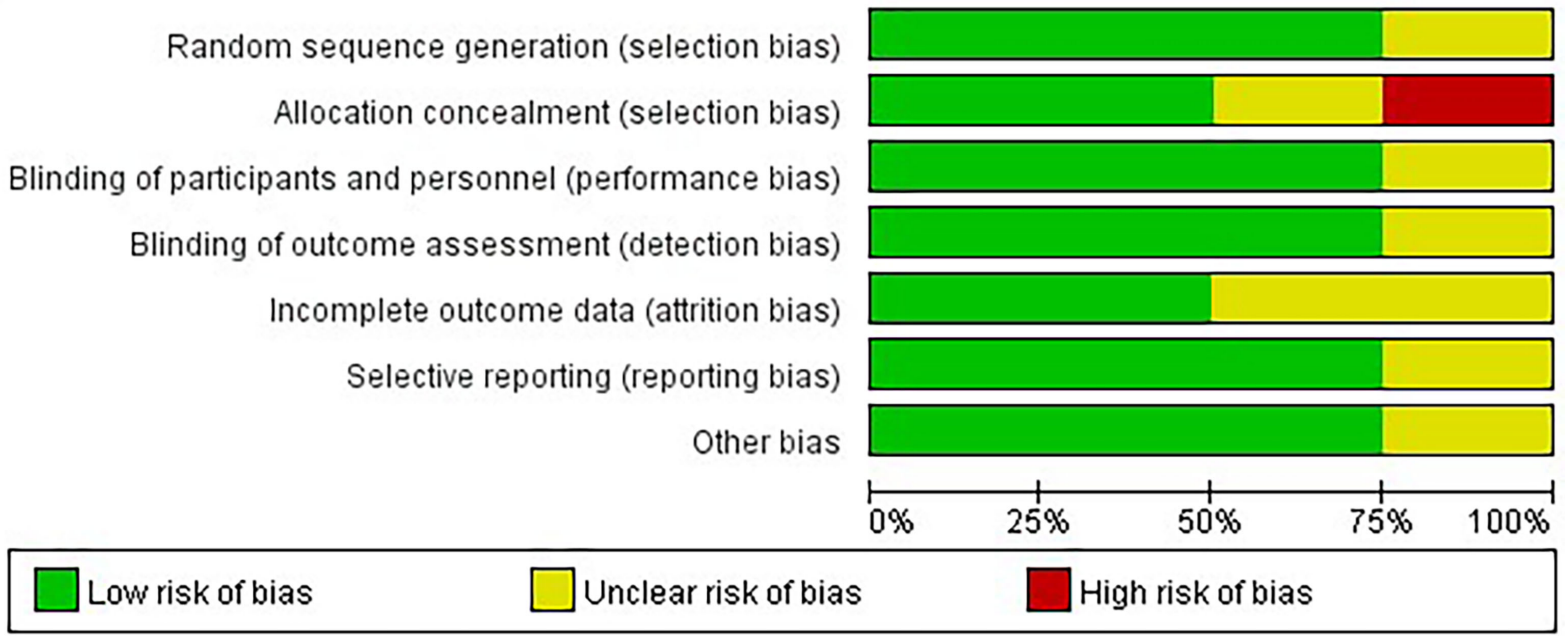
Quality assessment of included studies.

Four studies were conducted in 2018–2021. All studies were RCTs with a total of 244 more participants (in the treatment group, 120 participants used empagliflozin, while in the control group, 107 participants used a placebo in the control group and 20 participants used glimepiride), all patients were diagnosed with NAFLD (three of the study patients had comorbid diabetes and one was nondiabetic), two of the included studies were conducted in Iran, whereas the remaining two were conducted in India, China, and the duration of interventions varied from 20 to 24 weeks (The study’s attributes are detailed in **Supplementary Excel**). Four RCTs were parallel-group studies and articles of generally moderate and high quality based on the Cochrane Risk of Bias tool. The quality evaluation results of the four RCTs are delineated in **Figure 2**.

### The Effect of Empagliflozin on Liver Biological Indicators

**Figure 3:**
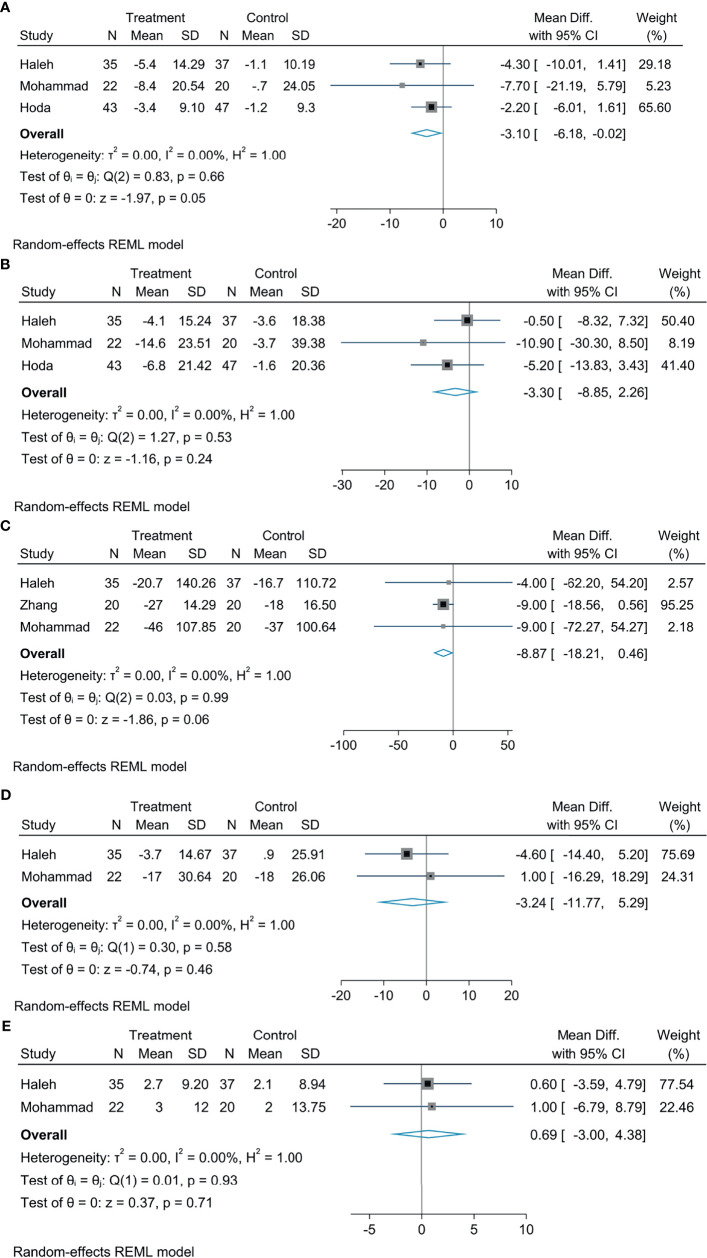
The effect of empagliflozin on AST **(A)**; the effect of empagliflozin on ALT **(B)** the effect of empagliflozin on TG **(C)**; the effect of empagliflozin on LDL **(D)**; the effect of empagliflozin on HDL **(E)**.

Meta-analysis concluded that empagliflozin was able to decrease AST levels among patients suffering from NAFLD compared with controls; the statistical difference was significant (MD: −3.10, 95% CI: −6.18 to −0.02, *p* = 0.05, *I*^2^ = 0%; **Figure 3A**). Empagliflozin was likely to reduce the levels of TG (MD: −8.87, 95% CI: −18.21 to 0.46, *p* = 0.06, *I*^2^ = 0%; **Figure 3B**) and ALT (MD: −3.30, 95% CI: −8.85 to 2.26, *p* = 0.24, *I*^2^ = 0%; **Figure 3C**), but the diversity was not of statistical significance. Compared with controls, there were no great diversity in HDL (MD: 0.69, 95% CI: −3.00 to 4.38, *p* = 0.71, *I*^2^ = 0%; **Figure 3D**) and LDL levels (MD: −3.24, 95% CI: −11.77 to 5.29, *p* = 0.46, *I*^2^ = 0%; **Figure 3E**).

### The Effect of Empagliflozin on Glycemic Indices

**Figure 4:**
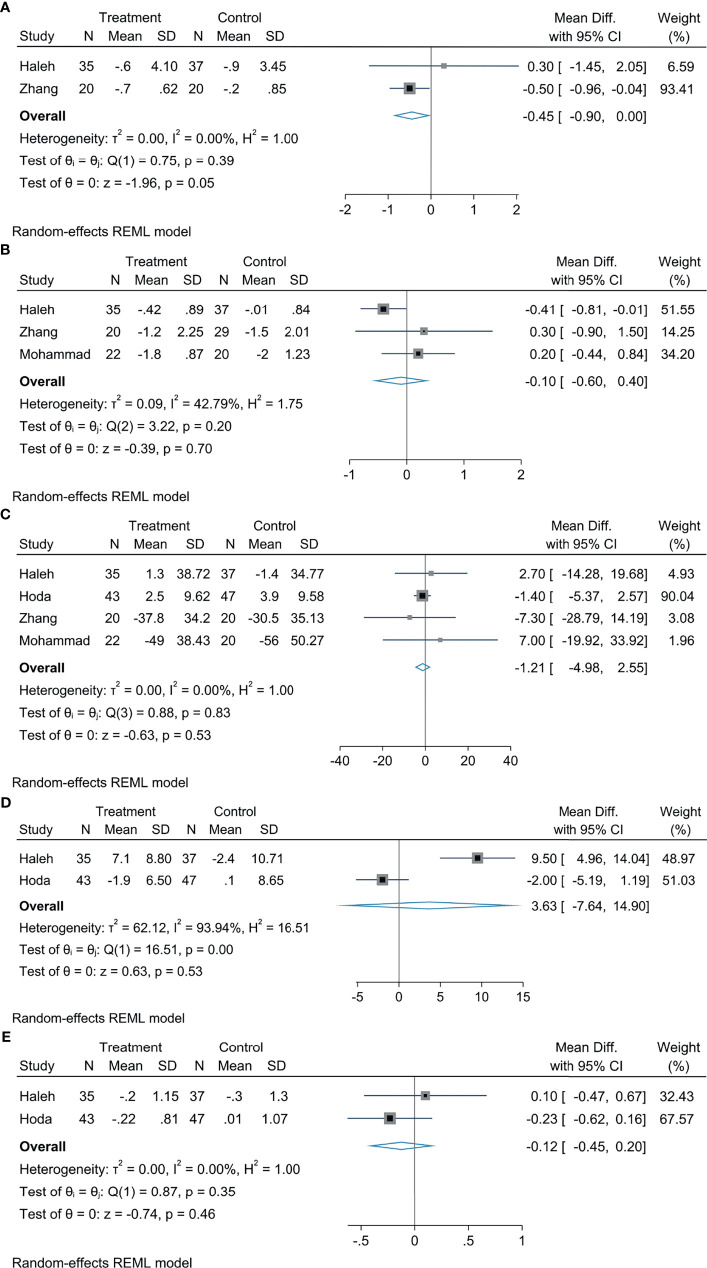
The effect of empagliflozin on HOMA-IR **(A)**; the effect of empagliflozin on HbA1c **(B)**; the effect of empagliflozin on FBS **(C)**; the effect of empagliflozin on insulin **(D)**; the effect of empagliflozin on HOMA2-IR **(E)**.

In comparison with the control group, empagliflozin treatment can attenuate HOMA-IR levels, the statistical difference was significant (MD: −0.45, 95% CI: −0.90 to 0.00, *p* = 0.05, *I*^2^ = 0%; **Figure 4A**), but there were no significant diversity in HbA1c (MD: −0.10, 95% CI: −0.60 to −0.40, *p* = 0.70, *I*^2^ = 42.79%; **Figure 4B**), FBS (MD: −1.21, 95% CI: −4.98 to 2.55, *p* = 0.53, *I*^2^ = 0%; **Figure 4C**), insulin (MD: 3.63, 95% CI: −7.64 to 14.90, *p* = 0.53, *I*^2^ = 90%; **Figure 4D**), and HOMA2-IR (MD: −0.12, 95% CI: −0.45 to 0.20, *p* = 0.46, *I*^2^ = 0%; **Figure 4E**).

### The Effect of Empagliflozin on Body Composition

**Figure 5:**
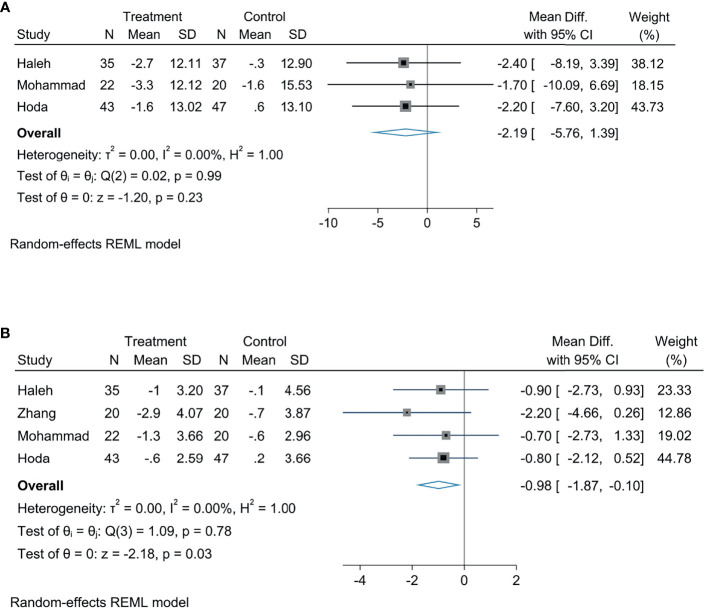
The effect of empagliflozin on weight **(A)**; the effect of empagliflozin on BMI **(B)**.

Our analysis demonstrated that empagliflozin obviously reduces the BMI in patients with NAFLD by comparing with controls, which had statistical differences (MD: −0.98, 95% CI: −1.87 to −0.10, *p* = 0.03, *I*^2^ = 0%; **Figure 5A**), empagliflozin was likely to reduce weight in patients with NAFLD by comparing with controls, but the statistical diversity was not great (MD: −2.19, 95% CI: −5.76 to 1.39, *p* = 0.23, *I*^2^ = 0%; **Figure 5B**).

### The Effect of Empagliflozin on Hepatic Steatosis and Fibrosis

**Figure 6:**
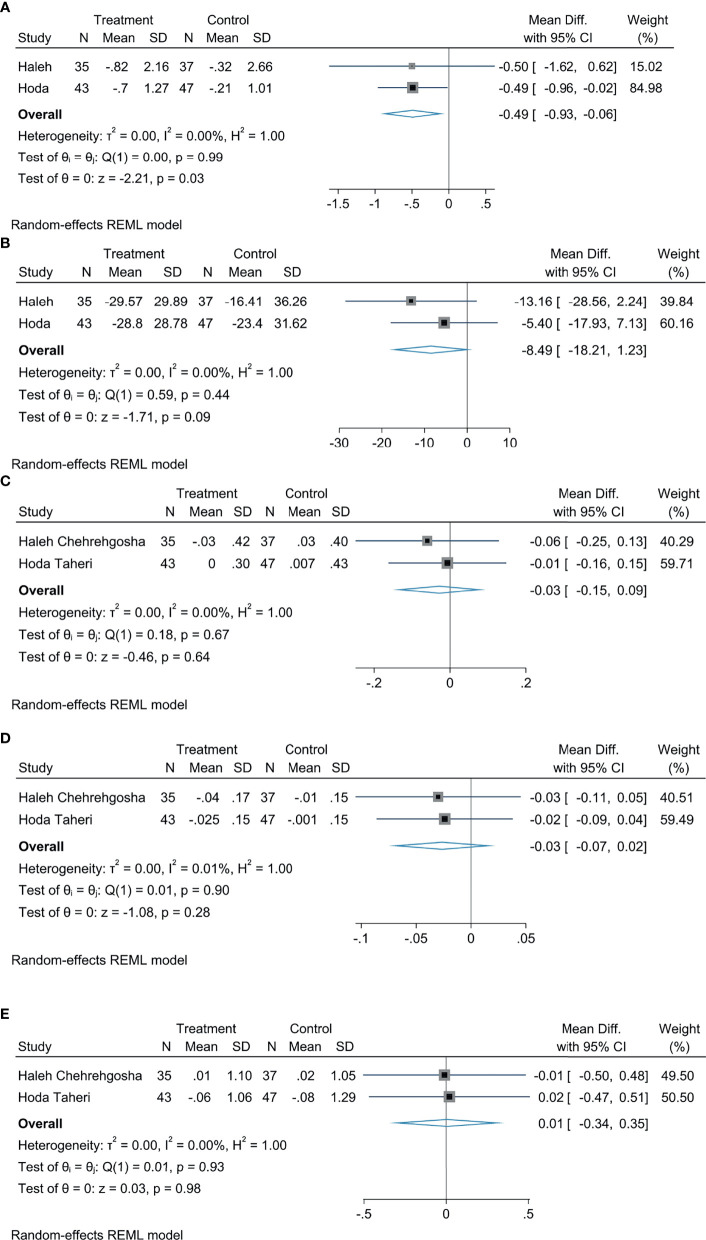
The effect of empagliflozin on LSM **(A)**; the effect of empagliflozin on CAP **(B)**; the effect of empagliflozin on FIB-4 **(C)**; the effect of empagliflozin on APRI **(D)**; the effect of empagliflozin on fasting plasma glucose NFS **(E)**.

Our results indicated that treatment with empagliflozin provoked a decreased LSM in patients with NAFLD by comparing with controls, with a statistically significant diversity (MD: −0.49, 95% CI: −0.93 to −0.06, *p* = 0.03, *I*^2^ = 0%; **Figure 6A**), and this analysis indicated that empagliflozin could reduce the CAP in patients with NAFLD, but there was no statistical significance (MD: −8.49, 95% CI: −18.21 to 1.23, *p* = 0.09, *I*^2^ = 0%; **Figure 6B**). According to the meta-analysis, empagliflozin did not greatly reduce the FIB-4 index (MD: −0.03, 95% CI: −0.15 to 0.09, *p* = 0.64, *I*^2^ = 0%; **Figure 6C**), APRI (MD: −0.03, 95% CI: −0.07 to 0.02, *p* = 0.28, *I*^2^ = 0%; **Figure 6D**), and NFS (MD: 0.01, 95% CI: −0.34 to 0.35, *p* = 0.98, *I*^2^ = 0%; **Figure 6E**) compared with controls.

### Adverse Events

Three RCTs (17, 18, 20) reported adverse events. The major adverse events consisted of nonspecific fatigue, arthralgia, mild hypoglycemia, mild fungal vaginal infections, mild allergic reactions, and urticaria. Overall, all the side effects were relieved after discontinuation of empagliflozin or appropriate local treatment. Mohammad et al. (17) covered that one patient discontinued the treatment after developing balanoposthitis. Haleh et al. (20) revealed that one patient discontinued the treatment owing to severe weakness and fatigue.

### Publication Bias

We only included 4 RCTs in this study; publication bias assessments were not conducted due to the insufficient number of articles.

## Discussion

This systematic review and meta-analysis of four RCTs was designed to assess the effectiveness and safety of empagliflozin in treating NAFLD patients. Most of these patients have T2DM. This analysis comprehensively assessed the impact of NAFLD patients in terms of glycemic control, body composition, biological indicators of liver function, and hepatic steatosis and fibrosis. The results of our research showed that empagliflozin had significant beneficial effects in decreasing body weight and improving liver function, liver fibrosis, and insulin resistance.

NAFLD has been the most prevalent etiology of chronic liver disease, which is a hot issue in recent research. NAFLD always have associations with changes in metabolic and nonmetabolic processes, such as dyslipidemia, oxidative stress, and insulin resistance. Therefore, attention should be paid to correcting all of these disturbances in the treatment of NAFLD. Current guidelines recommend changing life patterns as first-line therapy strategy for NAFLD. Nevertheless, no pharmacological therapy has been approved for NAFLD (21). Currently, studies have proven that liver histology in T2DM patients and non-T2DM patients with NASH confirmed by biopsy was improved by pioglitazone (3, 22). However, the adverse reactions of pioglitazone limit its use, including sodium and water leakage, weight gain, heart failure, and bone damage (3, 23). Moreover, glucagon-like peptide 1 receptor agonists have indicated beneficial effects in patients with T2DM and NAFLD (24). Several meta-analyses have demonstrated that SGLT2 inhibitors can lower liver enzymes, liver fat and improve body composition (25–27). The role of empagliflozin in NAFLD is not yet fully understood and deserves further investigation.

Empagliflozin is an SGLT2 inhibitor that attenuates blood glucose concentrations by restraining glucose reabsorption and accelerating glucose excretion. Additionally, empagliflozin also has exceptional profits, like cardiovascular protective actions (28), renal protection (29), lowering uric acid levels, and healing NAFLD-associated liver injury (30). In this meta-analysis, our results show reductions in liver function markers AST and ALT after empagliflozin treatment, although the change of ALT did not reach a statistical threshold. Changes in these parameters indicate an amelioration in NAFL or NASH (31). This may be due to the fact that empagliflozin can significantly improve NAFLD-related damage by enhancing autophagy by the liver macrophages and further restraining the inflammatory response (32).

The major underlying pathophysiologic mechanisms of NAFLD are insulin resistance and hepatic steatosis (33, 34). Evidence has demonstrated that patients treated with empagliflozin can improve hypothalamic insulin sensitivity and potentially benefit obesity and systemic metabolism (35). Studies have reported that empagliflozin can reverse fat and insulin resistance through lipid browning and macrophage activation (30). Improvements in obesity and insulin sensitivity may be associated with increased adiponectin levels, a bioactive protein known as adipokines (36). Empagliflozin promoted the replacement activation of macrophages in white adipose tissue and increased the plasma adiponectin level, resulting in obesity-induced inflammation and insulin resistance (30). Our results indicated that empagliflozin could enhance insulin sensitivity, as measured by HOMA-IR and HOMA2-IR, though the differences did not reach statistical significance. Empagliflozin has a beneficial effect on anthropometric parameters; BMI improved significantly in patients after empagliflozin treatment, but the change in weight was not of statistical significance.

Noninvasive biomarkers of liver fibrosis including the FIB-4 index, NFS, and APRI, were used to assess the severity degree of liver fibrosis (37), and the LSM was also adopted to evaluate liver fibrosis (38). End-stage hepatic fibrosis is a key prognostic biomarker for liver-associated outcomes and the total mortality (21). No effective treatment for hepatic fibrosis was found in the guidelines (39). This analysis demonstrated an obvious improvement in LSM scores after empagliflozin treatment; other markers of liver fibrosis including FIB-4 index, APRI, and NFS also depicted an amelioration with empagliflozin, to a large extent, although not statistically significant. This lack of statistically significant difference may be associated with the short duration of follow-up. The development of fibrosis may last for many years, with significant fibrosis reversal observed after long-term treatment (40). Studies with longer follow-up periods are required to verify the role of empagliflozin in the management of liver fibrosis in NAFLD patients. The specific mechanism of action of empagliflozin in liver fibrosis is not clear. However, it is hypothesized that empagliflozin achieves its antifibrosis effect by inhibiting proinflammatory cytokines such as IL-6, TNF-α, and MCP-1 (41)

NAFLD is currently a hot issue in research, and there are no other drugs in the guidelines that could be approved for treating NAFLD. At present, there is no meta-analysis of empagliflozin in the treatment of NAFLD. As we all know, this is the first meta-analysis to evaluate the role of empagliflozin in NAFLD. The advantage of this analysis is to comprehensively assess the roles of empagliflozin in glycemic control, body composition, levels of biological indicators of liver injury, and liver steatosis and fibrosis among patients with T2DM or non-T2DM combined with NAFLD. However, our study has several limitations: (a) Due to the limited large clinical trials, only a few RCTs were eligible, and only four RCTs were included in this paper. Most of the RCTs had small sample sizes, so the results are not significant. (b) The follow-up period of the included studies was short, and the duration was less than 6 months. There is no evidence of additional histological benefits from empagliflozin treatment for more than 6 months. The long-term prognosis and safety of empagliflozin remains unclear, so further research is required. (c) Liver biopsy as a gold standard for evaluating NAFLD (3). The included articles were not based on liver biopsy but relied on ultrasound and proton density fat fraction to diagnose NAFLD.

## Conclusions

In conclusion, our results suggest that empagliflozin significantly reduces the BMI, HOMA-IR, CAP score, and LSM score of both diabetic and nondiabetic NAFLD patients. However, the beneficial effects of empagliflozin did not achieve statistical significance in terms of body weight, FBS, HbA1C, LDL, HDL, TG, ALT, AST, insulin, HOMA2-IR, NFS, FIB-4 index, and APRI. Thus, more RCTs with longer duration and larger sample sizes are required to decide the roles of empagliflozin in patients with NAFLD and to establish adequate guidelines for clinical practice.

## Data Availability Statement

The original contributions presented in the study are included in the article/**Supplementary Material**. Further inquiries can be directed to the corresponding author.

## Author Contributions

YZ conducted a literature search, information extraction and information analysis and wrote the manuscript. XL extracted the data and reviewed the manuscript. XW and HZ were involved in review. All authors made contributions to the article and approved the submitted version.

## Funding

The Yunnan Provincial Science and Technology Department Project Fund of China (2017FH001-095) funded this study.

## Conflict of Interest

The authors declare that the research was conducted in the absence of any commercial or financial relationships that could be construed as a potential conflict of interest.

## Publisher’s Note

All claims expressed in this article are solely those of the authors and do not necessarily represent those of their affiliated organizations, or those of the publisher, the editors and the reviewers. Any product that may be evaluated in this article, or claim that may be made by its manufacturer, is not guaranteed or endorsed by the publisher.
