# Supplementary material for: Efficacy and Safety of Empagliflozin on Nonalcoholic Fatty Liver Disease: A Systematic Review and Meta-Analysis
Source: Front Endocrinol (Lausanne). 2022 Feb 24;13:836455. doi: 10.3389/fendo.2022.836455 (PMC8908261; doi:10.3389/fendo.2022.836455)
Supplement: Supplementary file 1 [file Table_1.docx]

Supplementary Material 1

**pubmed Search Strategy**

1. (((((((((((((Non-alcoholic fatty liver Disease[MeSH Terms]) OR (Non alcoholic Fatty Liver Disease[Title/Abstract])) OR (NAFLD[Title/Abstract])) OR (Nonalcoholic Fatty Liver Disease[Title/Abstract])) OR (Fatty Liver, Nonalcoholic[Title/Abstract])) OR (Fatty Livers, Nonalcoholic[Title/Abstract])) OR (Liver, Nonalcoholic Fatty[Title/Abstract])) OR (Livers, Nonalcoholic Fatty[Title/Abstract])) OR (Nonalcoholic Fatty Liver[Title/Abstract])) OR (Nonalcoholic Fatty Livers[Title/Abstract])) OR (Nonalcoholic Steatohepatitis[Title/Abstract])) OR (Nonalcoholic Steatohepatitides[Title/Abstract])) OR (Steatohepatitides, Nonalcoholic[Title/Abstract])) OR (Steatohepatitis, Nonalcoholic[Title/Abstract])
2. ((((Empagliflozin[Supplementary Concept]) OR (BI 10773[Title/Abstract])) OR (BI10773[Title/Abstract])) OR (BI-10773[Title/Abstract])) OR (Jardiance[Title/Abstract])
3. ((randomized controlled trial[Publication Type]) OR (randomized[Title/Abstract])) OR (placebo[Title/Abstract])
4. #1 and #2 and #3

**Web of Science Search Strategy**

1. TS=(Non alcoholic Fatty Liver Disease OR NAFLD OR Nonalcoholic Fatty Liver DiseaseOR Fatty Liver, Nonalcoholic OR Fatty Liver*, Nonalcoholic OR Liver, Nonalcoholic Fatty OR Liver*, Nonalcoholic Fatty OR Nonalcoholic Fatty Liver OR Nonalcoholic Fatty Liver* OR Nonalcoholic Steatohepatitis OR Nonalcoholic Steatohepatitide* OR Steatohepatitide*, Nonalcoholic OR Steatohepatitis, Nonalcoholic)
2. TS=(Empagliflozin OR BI 10773 OR BI10773 OR BI-10773 OR Jardiance)
3. TS=(random* controlled trial OR random* OR placebo)
4. #1 AND #2 AND #3

**Cochrane Search Strategy**

1. (empagliflozin:ti,ab,kw) or (BI 10773:ti,ab,kw) or (BI10773:ti,ab,kw) or (BI-10773 Jardiance:ti,ab,kw)
2. MeSH descriptor:[Non-alcoholic Fatty Liver Disease] explode all trees
3. (Non alcoholic Fatty Liver Disease:ti,ab,kw) or (NAFLD:ti,ab,kw) or (Nonalcoholic Fatty Liver Disease:ti,ab,kw) or (Fatty Liver, Nonalcoholic:ti,ab,kw) or (Fatty Livers, Nonalcoholic:ti,ab,kw) or (Liver, Nonalcoholic Fatty:ti,ab,kw) or (Liver, Nonalcoholic Fatty:ti,ab,kw) or (Nonalcoholic Fatty Liver:ti,ab,kw) or (Nonalcoholic Fatty Livers:ti,ab,kw) or (Nonalcoholic Steatohepatitis:ti,ab,kw) or (Nonalcoholic Steatohepatitides:ti,ab,kw) or (Steatohepatitides, Nonalcoholic)
4. (randomized Controlled Trial:pt) or (randomized:ti,ab,kw) or (placebo:ti,ab,kw)
5. #1 and #3 and #4

**Embase Search Strategy**

1. 'empagliflozin'/exp
2. ‘BI 10773’:ti,ab or ‘BI10773’:ti,ab or ‘BI-10773’:ti,ab or ‘Jardiance’:ti,ab
3. #1 and #2
4. 'nonalcoholic fatty liver'/exp
5. ‘Non alcoholic Fatty Liver Disease’:ti,ab or ‘NAFLD’:ti,ab or ‘Nonalcoholic Fatty Liver Disease’:ti,ab or ‘Fatty Liver, Nonalcoholic’:ti,ab or ‘Fatty Livers, Nonalcoholic’:ti,ab or ‘Liver, Nonalcoholic Fatty’:ti,ab or ‘Livers, Nonalcoholic Fatty’:ti,ab or ‘Nonalcoholic Fatty Liver’:ti,ab or ‘Nonalcoholic Fatty Livers’:ti,ab or ‘Nonalcoholic Steatohepatitis’:ti,ab or ‘Nonalcoholic Steatohepatitides’:ti,ab or ‘Steatohepatitides, Nonalcoholic’:ti,ab or ‘Steatohepatitis, Nonalcoholic’:ti,ab
6. #4 and #5
7. ' randomized Controlled Trial '/exp
8. 'randomized ':ti,ab OR 'placebo':ti,ab
9. #7 and #8
10. #3 and #6 and #9
